# Dual-faced circRNAs: orchestrating immunosuppression and activation in the lung cancer microenvironment

**DOI:** 10.3389/fcell.2025.1706393

**Published:** 2025-12-04

**Authors:** Junyao Li, Lin Zhang, Yicun Wang, Chunyan Li

**Affiliations:** 1 Respiratory and Critical Care Medicine Department of the Second Hospital of Jilin University, Changchun, China; 2 Department of Medical Research Center, Second Hospital of Jilin University, Changchun, China; 3 Department of Respiratory and Critical Care Medicine, Qilu Hospital (Qingdao), Cheeloo College of Medicine, Shandong University, Qingdao, China

**Keywords:** circular RNAs, immune microenvironment, immune checkpoint, immunotherapy resistance, CAR-T therapy

## Abstract

Circular RNAs (circRNAs), a class of non-coding RNAs characterized by covalently closed-loop structures, have emerged as key regulators in the tumor immune microenvironment (TIME) of lung cancer, owing to their high stability, tissue-specific expression, and multidimensional regulatory capabilities. This review systematically synthesizes the latest research progress and elucidates the processes by which circRNAs regulate the functional states of immune cells in the TIME through diverse molecular mechanisms, including acting as competing endogenous RNAs (ceRNAs) to sequester microRNAs (miRNAs), interacting with RNA-binding proteins (RBPs), and in some cases, encoding functional polypeptides. CircRNAs possess bidirectional regulatory capacities: they can promote tumor immune evasion by modulating the expression of immune checkpoint molecules, influencing the infiltration and activity of effector immune cells (e.g., CD8^+^ T cells), recruiting immunosuppressive cells (e.g., regulatory T cells and M2-type macrophages), and regulating immune signaling pathways; meanwhile, they can also activate antitumor immune responses. Furthermore, the review explores the potential of circRNAs as liquid biopsy biomarkers for lung cancer diagnosis and prognosis, as well as their translational prospects in therapeutic strategies including vaccines, circRNA-enhanced CAR-T therapy, and formulations encoding immunomodulatory factors. Despite challenges such as complex mechanisms, low delivery efficiency, and safety concerns, the development of multi-omics technologies, novel delivery systems, and gene-editing tools provides directions for the development of precision therapies targeting circRNAs, which aim to reshape the lung cancer immune microenvironment and overcome immunotherapy resistance.

## 1 Introduction

Lung cancer remains the most lethal malignancy globally, with non-small cell lung cancer (NSCLC) accounting for the majority of cases (Ramos-Esquivel, 2022). Although immunotherapy, particularly immune checkpoint inhibitors (ICIs), has transformed the treatment paradigm for advanced lung cancer, its overall response rate remains suboptimal, and primary or secondary resistance is frequently observed, leading to poor patient outcomes. The heterogeneity and immunosuppressive nature of the tumor immune microenvironment (TIME) (Teng et al., 2025) are fundamental factors limiting the efficacy of immunotherapy. The TIME is a dynamic ecosystem composed of tumor cells, a diverse array of immune cells (including T cells, B cells, natural killer cells, macrophages, and dendritic cells) (Li et al., 2022a), stromal cells, the extracellular matrix, and an intricate network of signaling molecules. Deciphering the regulatory mechanisms within the TIME and identifying novel targets to reverse its immunosuppressive state are crucial for improving the effectiveness of lung cancer immunotherapy.

In recent years, circular RNAs (circRNAs) (Babin et al., 2021)—generated via back-splicing of precursor mRNAs (pre-mRNAs) to form covalently closed-loop non-coding RNA loops—have become a focal point in life sciences research. Their structural stability, tissue- and temporal-specific expression profiles, and evolutionary conservation have attracted significant attention. Accumulating evidence challenges the conventional view of circRNAs as mere splicing byproducts, instead revealing their extensive involvement in the pathogenesis of various diseases, including cancer. Through mechanisms such as miRNA sponging (Jie et al., 2022), RBP interactions (Khoroshkin et al., 2024), regulation of gene transcription (Henninger and Young, 2024), and even peptide translation, circRNAs play critical roles in disease development.

In the context of lung cancer, circRNA expression is markedly dysregulated. Growing studies demonstrate that these circRNA molecules act as key regulators of the TIME. By precisely modulating the expressions of immune checkpoint proteins (e.g., PD-L1) (Wang et al., 2022), influencing the secretion of cytokines and chemokines, and orchestrating the recruitment, differentiation, and function of immune cells (including effector T cells, Treg cells, tumor-associated macrophages, and myeloid-derived suppressor cells (MDSCs)) (Lan et al., 2021), circRNAs shape the delicate balance between immunosuppression and immune activation within the TIME. However, most of these regulatory mechanisms are initially validated *in vitro* (e.g., NSCLC cell lines like A549 or H1299), and *in vivo* verification is still lacking for many circRNAs (e.g., circSHKBP1 and circ_0002483). This gap limits the reliability of their therapeutic potential as *in vitro* results may not fully recapitulate the complex TIME in human lung cancer. A thorough understanding of the molecular mechanisms underlying circRNA-mediated regulation of the lung cancer TIME is not only essential for uncovering new insights into tumor immune evasion but also provides a solid foundation for the development of circRNA-based diagnostic biomarkers (Wu et al., 2023), prognostic tools, and innovative immunotherapeutic strategies.

This review aims to comprehensively summarize the central roles and bidirectional regulatory functions of circRNAs in reprogramming the lung cancer TIME, dissect their molecular mechanisms of action, and discuss the challenges and future directions in translating these findings into clinical applications.

## 2 Functional mechanisms of circular RNAs

**FIGURE 1:**
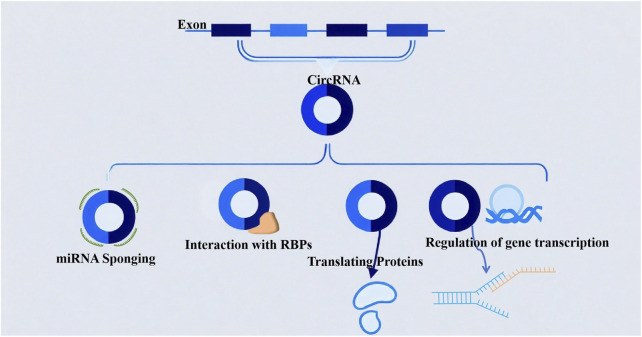
The biological functions of circular RNAs (circRNAs): (1) acting as miRNA sponges to sequester microRNAs; (2) modulating protein interactions through binding with RNA-binding proteins (RBPs); (3) possessing protein-coding potential; (4) participating in the regulation of gene transcription.

Circular RNAs (circRNAs) are primarily generated through back-splicing: during the splicing of precursor mRNAs (pre-mRNAs) in the nucleus, a downstream splice donor site covalently links to an upstream splice acceptor site, forming a closed circular structure (Ma et al., 2022). This splicing event is tightly regulated by RNA-binding proteins (RBPs) and can be dynamically modulated by gene expression levels, cell differentiation status, and extracellular environmental cues (e.g., hypoxia and inflammation). Owing to their covalently closed-loop structure, circRNAs exhibit higher stability than linear RNAs and display distinct tissue- and cell-type-specific expression patterns. Their functional mechanisms in gene regulation are diverse, including the following key modes (Figure 1).

### 2.1 miRNA sponging

CircRNAs can act as competing endogenous RNAs (ceRNAs) by sequestering specific microRNAs (miRNAs) via complementary binding sites, thereby relieving the translational repression of the miRNAs’ target mRNAs. A classic example is CDR1as (also known as ciRS-7) (Shao et al., 2023), which contains more than 70 conserved binding sites for miR-7; by sponging miR-7, CDR1as upregulates the expression of miR-7 target genes involved in cell proliferation and apoptosis (Scoyni et al., 2024).

### 2.2 Interaction with RNA-binding proteins (RBPs)

CircRNAs can directly bind to RBPs to modulate their activity, subcellular localization, or interaction with target nucleic acids, thereby regulating gene transcription or mRNA stability. Notably, recent studies highlight the role of circRNA–RBP interactions in lung cancer immunosuppression: for instance, circEPSTI1 binds to the RBP HuR to stabilize PD-L1 mRNA, thereby enhancing PD-L1 expression on NSCLC cells and promoting immune evasion (Khan et al., 2022). In NSCLC, circDENND2D interacts with the RBP HuR (rather than miR-130b-3p (Chen et al., 2023), as previously inaccurately reported) to prevent HuR from stabilizing STK11 mRNA. Reduced STK11 expression subsequently inhibits the transcription and translation of PD-L1, thereby reversing PD-L1-mediated immune escape. CircMET binds to the RBP YBX1 in lung adenocarcinoma, blocking YBX1’s interaction with IL-2 mRNA and suppressing IL-2 translation, which further illustrates how circRNA-RBP interactions disrupt cytokine production and T cell activation (Khan et al., 2024). Additionally, a study reinforces the diversity of circRNA-RBP functions, noting that lung cancer circRNAs can interfere with RBP-dependent interferon signaling—thereby extending beyond splicing and translation control to immune-related pathway regulation.

### 2.3 Translating proteins

Although traditionally classified as non-coding RNAs, a subset of circRNAs (e.g., those containing internal ribosomal entry sites [IRES]) can be translated into functional short peptides. These circRNA-encoded peptides have been implicated in regulating cellular signaling pathways (e.g., MAPK/ERK) and metabolic processes (Papatsirou et al., 2021), contributing to tumorigenesis and immune cell function.

### 2.4 Regulation of gene transcription

Certain circRNAs, such as exon–intron circRNAs (EIciRNAs) and intronic circRNAs (ciRNAs), retain intronic sequences that enable interaction with U1 small nuclear ribonucleoproteins (snRNPs) or RNA polymerase II (Zhang S. et al., 2021). For example, EIciRNAs localized in the nucleus can bind to U1 snRNPs and recruit them to the promoter region of host genes, thereby enhancing gene transcription (e.g., circEIF3J promotes EIF3J expression to support lung cancer cell proliferation) (Wei et al., 2020).

## 3 The lung cancer immune microenvironment

The TIME in lung cancer is a dynamic and highly heterogeneous ecosystem composed of tumor cells, immune cells, stromal cells (e.g., fibroblasts and endothelial cells), the extracellular matrix, and a complex network of cytokines and chemokines. This microenvironment critically influences tumor progression, metastasis, and response to immunotherapy. Anti-tumor immune cells—including CD8^+^ T cells, NK cells, M1-type macrophages, and dendritic cells (Calmeiro et al., 2020)—contribute to tumor suppression by directly killing tumor cells or activating adaptive immunity. In contrast, pro-tumor immune cells such as M2-type macrophages, regulatory T cells (Tregs), and MDSCs (Feng et al., 2025; Flores-Borja and Blair, 2022; Khan et al., 2020) suppress immune responses by secreting inhibitory factors (e.g., TGF-β and IL-10) or depleting essential nutrients. Cytokines and chemokines (e.g., CCL5, CXCL10, and IFN-γ) act as signaling molecules that coordinate intercellular communication, regulating immune cell recruitment and function (Liao et al., 2023; Yu et al., 2020).

**FIGURE 2:**
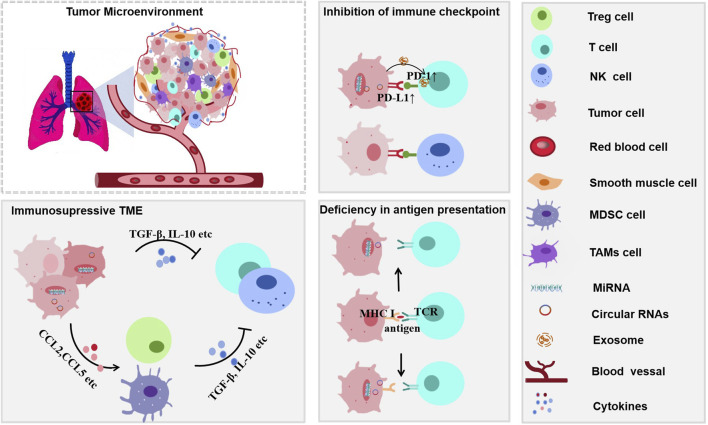
Mechanisms of immune evasion in lung cancer through an immunosuppressive tumor microenvironment (TIME). The immunosuppressive TIME facilitates immune escape via multiple pathway. Immunosuppressive cytokines such as TGF-β and IL-10 inhibit antitumor immunity. Upregulation of PD-L1 on tumor cells leads to checkpoint inhibition, while impaired antigen presentation is symbolized by the disrupted MHC I–TCR interaction. Multiple cell types contribute to the immunosuppressive milieu, further promoting tumor immune evasion.

To evade immune surveillance, lung cancer cells employ multilayered strategies (Figure 2): they directly secrete immunosuppressive factors (e.g., TGF-β and IL-10) to impair CD8^+^ T and NK cell activity and regulate the quantity and function of Treg cells; lung cancer cells release signals (e.g., CCL2) to recruit immunosuppressive cells (e.g., CCR2+ tumor-associated macrophages[TAMs], MDSCs, and Th2) to create an immunosuppression microenvironment that contributes to tumor progression (Ni et al., 2023; Bergerud et al., 2024); and critically, exploit immune checkpoint pathways. By overexpressing PD-L1 (which binds to PD-1 on T cells), they induce T cell exhaustion. They exploit CTLA-4, which competitively binds to B7 molecules on antigen-presenting cells, thereby inhibiting the activation and proliferation of T cells (Zhang et al., 2024; John et al., 2022). Additionally, lung cancer cells downregulate the expression of surface MHC class I molecules/tumor antigens to evade recognition by T cells (Lei et al., 2022). Together, these mechanisms enable the tumor to sustain progression under immune pressure.

## 4 Bidirectional regulatory role of CircRNA in the lung cancer tumor immune microenvironment

The TIME of lung cancer is a highly complex and dynamic ecosystem. Its composition and functional status directly influence the response to immunotherapy and clinical outcomes (Shi et al., 2025). CircRNAs—a class of non-coding RNAs with covalently closed-loop circular structures—have emerged as key “bidirectional regulators” in shaping the lung cancer TIME. A critical analysis of their functions reveals that diverse circRNAs frequently converge onto a limited set of pivotal signaling axes to exert their effects. Notably, a dominant mechanism is the targeting of the PD-1/PD-L1 checkpoint pathway by numerous circRNAs (e.g., circIGF2BP3 and circRNA-002178), which act as ceRNAs to enhance checkpoint expression and drive T cell exhaustion. Another recurring theme is the orchestration of T cell chemotaxis, where circRNAs such as circCELF1 and circFGFR1 dysregulate chemokine signaling (e.g., suppressing CXCL10 and upregulating CXCR4) to inhibit CD8^+^ T cell infiltration and foster an immune-excluded phenotype. Conversely, immunostimulatory circRNAs like cEMSY can activate innate immune sensing via the cGAS-STING pathway to promote antigen presentation and T cell priming. Due to their high stability, cell type-specific expression, and diverse regulatory functions, circRNAs can not only promote immune escape and tumor progression through multiple mechanisms but also include subsets that exert tumor-suppressive and immunostimulatory effects. Although studies in other cancer types have revealed regulatory roles of circRNAs in NK cell function, this area remains unexplored in lung cancer and warrants further investigation.

### 4.1 Mechanisms of promoting immune suppression and tumor progression

#### 4.1.1 Direct targeting of immune checkpoint pathways

**FIGURE 3:**
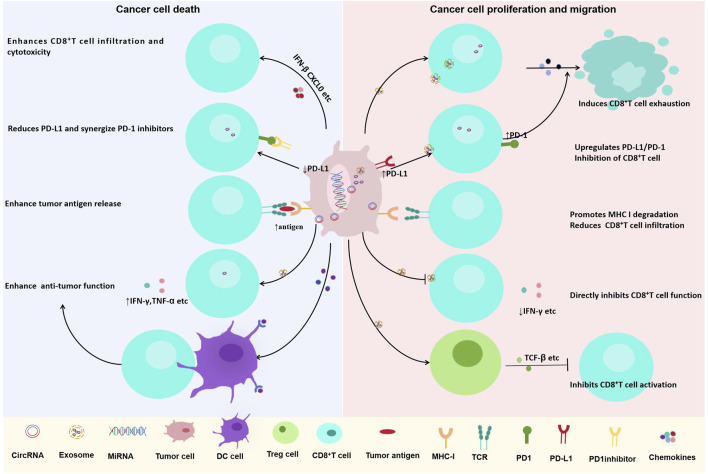
Dual roles of circRNAs in regulating CD8^+^ T cell-mediated anti-tumor immunity in lung cancer. CircRNAs play context-dependent roles in modulating CD8^+^ T cell function and tumor immune responses. (Left) Certain circRNAs enhance anti-tumor immunity by promoting CD8^+^ T cell infiltration and cytotoxicity, downregulating PD-L1 expression, increasing tumor antigen release, and enhancing IFN-γ and TNF-α production. (Right) Other circRNAs contribute to immune evasion by inducing CD8^+^ T cell exhaustion, upregulating PD-L1/PD-1 checkpoint expression, promoting MHC-I degradation, reducing CD8^+^ T cell infiltration, and directly inhibiting CD8^+^ T cell function through immunosuppressive cytokines such as TGF-β.

CircRNAs orchestrate immune checkpoint expression through multifaceted mechanisms, ultimately leading to T cell exhaustion. A prominent strategy is the post-translational stabilization of PD-L1 (Table 1). For example, circIGF2BP3 functions as a ceRNA for miR-328-3p and miR-3173-5p (Liu et al., 2021), culminating in the stabilization of the PD-L1 protein via the OTUB1 deubiquitinase. Beyond regulating stability, circRNAs can also coordinate the bidirectional expression of immune checkpoints across different cell types within the TIME. A paradigm is circRNA-002178, which operates in a dual capacity: it directly upregulates PD-L1 expression in tumor cells by sequestering miR-34a (Yang et al., 2020; Wang et al., 2023); and is subsequently packaged into exosomes to be transferred to CD8^+^ T cells, where it suppresses miR-28-5p to elevate PD-1 expression. This coordinated action efficiently hyperactivates the PD-1/PD-L1 axis (Yue et al., 2020; Pauken et al., 2025) (Figure 3).

**TABLE 1:** CircRNAs directly regulating PD-L1/immune checkpoints to drive immune escape in immune escape in lung cancer.

circRNA name	Subtype	Core mechanism	Immune effect	Clinical significance	References
circIGF2BP3	NSCLC	Sponges miR-328-3p/miR-3173-5p → upregulates PKP3 → stabilizes OTUB1 → increases PD-L1 protein abundance	Inhibits CD8^+^T cell function, induces CD8^+^T cell exhaustion, and reduces CD8^+^T cell infiltration	High expression indicates anti-PD-1 therapy resistance and is associated with poor prognosis	Liu et al. (2021)
circRNA-002178	Lung Adenocarcinoma	1. Absorbs miR-34a in tumor cells → upregulates PD-L1; 2. Enters CD8^+^T cells via exosomes, sponges miR-28-5p → upregulates PD-1	Bidirectionally activates PD-1/PD-L1 pathway and induces CD8^+^T cell exhaustion	Serves as a diagnostic biomarker for lung adenocarcinoma; high expression correlates with poor immunotherapy response	Wang et al. (2020a)
hsa_circ_0000190	NSCLC	1. Targets miR-142-5p and miR1299, induces PD-L1 expression; 2. Promotes secretion of soluble PD-L1 (sPD-L1)	sPD-L1 interferes with T cell activation and inhibits CD8^+^T cell infiltration	High expression is associated with adverse reactions to immunotherapy and serves as a resistance biomarker	Luo et al. (2021)
circ_0000284	NSCLC	Sponges miR-377-3p→ relieves inhibition on PD-L1	Upregulates PD-L1, and promotes tumor cell migration and invasion	Associated with malignant progression of NSCLC (e.g., lymph node metastasis) and therapy resistance	Li et al. (2020a)
circ-HSP90A	NSCLC	Sponges miR-424-5p → upregulates PD-L1	Induces CD8^+^T cell apoptosis and inhibits CD8^+^T cell function	Associated with insufficient CD8^+^T cell infiltration and anti-PD-1 therapy resistance	Lei et al. (2023)
hsa_circ_0068252	NSCLC	Sponges miR-1304-5p → upregulates PD-L1	Promotes immune escape and is associated with cisplatin resistance	Serves as an independent risk factor for poor prognosis; predicts resistance to combined therapy	Li et al. (2022c)
circCHST15	NSCLC	Sponges miR-155-5p/miR-194-5p → upregulates PD-L1	Upregulates PD-L1; expression is positively correlated with tumor stage	Can be used as a biomarker for immune staging and efficacy prediction	Yang et al. (2021)
circ_001678	NSCLC	Sponges miR-326 → upregulates ZEB1 → activates PD-1/PD-L1 transcription	Promotes CD8^+^T cell apoptosis and reduces CD8^+^T cell proportion	Associated with PD-1 inhibitor resistance and shortened overall survival	Tian et al. (2022)
circ_0014235	NSCLC	Sponges miR-146b-5p → upregulates YAP → indirectly promotes PD-L1 transcription	Promotes immune escape and induces gefitinib resistance	Indicates poor response to targeted therapy combined with immunotherapy in EGFR-mutant patients	Niu et al. (2022)
circBIRC6	NSCLC	Sponges miR-217, → may enhance PD-L1 function	Impairs T cell-mediated tumor cell clearance	Potential immunotherapeutic target; mechanism needs verification based on lung cancer subtypes	Ni et al. (2022)

#### 4.1.2 Impairing effector immune cell function or infiltration

Another subset of circRNAs systemically suppresses anti-tumor immunity by interfering with T cell recruitment, activation, proliferation, and antigen presentation (Tables 2–4). For example, circCELF1 sequesters miR-491-5p (Ge et al., 2021), relieving its inhibition of EGFR. Aberrant EGFR activation transcriptionally represses the key T cell chemokine CXCL10 (Yan et al., 2023), hindering CD8^+^ T cell migration into tumor sites.

**TABLE 2:** CircRNAs inhibiting effector immune cell (CD8^+^T Cell) function/infiltration.

circRNA name	Subtype	Core mechanism	Immune effect	Clinical significance	References
circCELF1	NSCLC	Sponges miR-491-5p → upregulates EGFR → inhibits CXCL10 secretion	Restricts CD8^+^T cell recruitment and impairs CD8^+^T cell activation	Associated with anti-PD-1 therapy failure and shortened progression-free survival	Ge et al. (2021)
circASCC3	NSCLC	Sponges miR-432-5p → upregulates C5a → activates C5a/C5aR axis	Induces CD8^+^T cell exhaustion and promotes M2 macrophage polarization	Key driver of immunotherapy resistance; high expression indicates ineffective treatment	Gao et al. (2022b)
circFGFR1	NSCLC	Sponges miR-381-3p → upregulates CXCR4 → interferes with T cell chemotaxis	Reduces CD8^+^T cell infiltration and forms “immune-cold tumors”	Associated with low CD8^+^T cell proportion and poor immunotherapy response	Zhang et al. (2019)
circUSP7	NSCLC	Sponges miR-934 → upregulates SHP2 → inhibits TCR signaling pathway	Directly inhibits CD8^+^T cell function (reduced IFN-γ secretion)	Serves as a biomarker for immunotherapy resistance and correlates with shortened overall survival	Chen et al. (2021a)
circRUNX1	NSCLC	Sponges miR-4739 → upregulates PCSK9 → promotes MHC I degradation	Reduces antigen presentation ability and CD8^+^T cell infiltration	Associated with complete non-response to immunotherapy	Zhang et al. (2021b)

**TABLE 3:** CircRNAs recruiting/inducing immunosuppressive cells (Treg and M2-type macrophages).

circRNA name	Subtype	Core mechanism	Immune effect	Clinical significance	References
circ_0004140	Lung Adenocarcinoma	Sponges miR-1184 → upregulates CCL22 (Treg chemokine)	Recruits a large number of Tregs and inhibits effector T cell function	Associated with anti-PD-1 therapy resistance and increased tumor recurrence rate	Liu et al. (2022)
circZNF451	Lung Adenocarcinoma	Forms a complex with TRIM56/FXR1 → activates ELF4-IRF4 pathway	Induces M2 macrophage polarization and promotes CD8^+^T cell exhaustion	Can predict PD-1 blockade therapy resistance and correlates with metastasis risk	Gao et al. (2022a)
circHIPK3	NSCLC	Activates circHIPK3/PTK2 pathway	Promotes MDSC differentiation into M2 macrophages	Associated with immunosuppression, increased tumor angiogenesis, and poor prognosis	Wang and Chen (2024)
hsa_circ_0003026	NSCLC	Mediates M2 macrophage polarization via the hsa-miR-1183/XRN2 axis	Increases M2 macrophage infiltration and forms immunosuppressive TME	Potential exosome-related immunotherapeutic target	Zhu et al. (2025)
circSHKBP1	NSCLC	Directly promotes M2 macrophage polarization (miRNA axis not clarified)	Recruits M2 macrophages and impairs CD8^+^T cell function	Associated with NSCLC migration, invasion, and immunosuppression	Chen et al. (2022)
circFARSA	NSCLC	Activates PTEN/PI3K/AKT pathway via exosomes → induces M2 polarization	Promotes M2 macrophage polarization and accelerates tumor metastasis	Can be used as a metastasis-related immunotherapeutic target	Chen et al. (2021b)

**TABLE 4:** CircRNAs regulating cytokines/signaling pathways to indirectly inhibit the immune response.

circRNA name	Subtype	Core mechanism	Immune effect	Clinical significance	References
circHMGB2	NSCLC	Interacts with miR-181a-5p → upregulates CARM1 → inhibits type I interferon response	Reduces IFN-I secretion; decreases tumor cell sensitivity to T cell-mediated killing	Enables tumor cells to escape immune clearance; indicates poor immunotherapy efficacy	Zhang et al. (2022)
hsa_circ_0003222	NSCLC	Sponges miR-527 → upregulates PIF1B	Promotes tumor stemness and inhibits type I interferon secretion	Drives NSCLC progression and induces anti-PD-1 therapy resistance	Li et al. (2021b)
hsa_circ_0020714	NSCLC	Sponges miR-30a-5p → upregulates SOX4	Inhibits DC cell maturation and promotes immune escape	Associated with immunotherapy resistance and shortened survival	Wu et al. (2022)
circ_0002483	Lung Adenocarcinoma	Regulates miR-125a-3p/CCL4-CCR5 axis	Interferes with T cell and macrophage chemotaxis; disrupts immune response	Associated with lung adenocarcinoma progression and immune escape; potential target	Wan et al. (2021)

In a parallel mechanism, circFGFR1 sponges miR-381-3p, resulting in the upregulation of CXCR4 expression (Zhang et al., 2019). This dysregulation disrupts chemotactic signaling and impairs T cell trafficking, fostering the development of immune-excluded (“cold”) tumor phenotypes.

Further contributing to T cell dysfunction, circUSP7 binds and inhibits miR-934, leading to sustained expression of SHP2 (Chen S. W. et al., 2021). Elevated SHP2 protein attenuates T cell receptor (TCR) signaling, suppresses the production of effector cytokines such as IFN-γ and TNF-α, and ultimately diminishes CD8^+^ T cell cytotoxicity (Figure 3). Collectively, these circRNAs undermine antitumor immunity through synergistic actions across multiple cellular and molecular tiers.

#### 4.1.3 Recruiting or inducing immunosuppressive cells to establish a suppressive microenvironment

Certain circRNAs promote the recruitment, differentiation, and activation of immunosuppressive cells (e.g., Tregs, M2 macrophages, and MDSCs) by modulating chemokine secretion and key signaling pathways. circ_0004140 upregulates the Treg-recruiting chemokine CCL22 expression (Liu et al., 2022), facilitating Treg accumulation and subsequent suppression of effector T cells via cell-contact inhibition and cytokine secretion (e.g., IL-10 and TGF-β). circZNF451 forms a complex with TRIM56 and FXR1 (Gao et al., 2022a), activating the ELF4–IRF4 axis and driving M2 polarization of TAMs. circFARSA is secreted via exosomes and internalized by TAMs (Chen T. et al., 2021), where it activates the PTEN/PI3K/AKT pathway. This promotes M2 polarization, leading to upregulation of anti-inflammatory factors, angiogenesis promotion, and suppression of adaptive immunity through molecules such as VEGF and IL-10.

### 4.2 Mechanisms of enhancing anti-tumor immunity

In contrast to immunosuppressive circRNAs, another group has been identified as potent activators of antitumor immunity, capable of reversing immunosuppressive cues within the tumor microenvironment (Table 5). These immunostimulatory circRNAs provide a compelling molecular basis for developing novel RNA-based immunotherapies.

**TABLE 5:** CircRNAs inhibiting lung cancer development by regulating tumor immune microenvironment.

Name	Core mechanism	Immune regulatory effect	Clinical relevance	Reference
circNDUFB2	1. Recruits TRIM25 to promote ubiquitination and degradation of IGF2BPs, blocking stabilization of MYC and MMP2; 2. Binds RIG-I to activate RIG-I-MAVS pathway, inducing release of IFNβ and CXCL10	1. Enhances CD8^+^T cell infiltration and cytotoxicity; 2. Inhibits HLA-F/LILRB1- mediated immune escape, improving T cell recognition and killing ability	1. Low expression associated with reduced CD8^+^T cell infiltration and poor prognosis in NSCLC; 2. Overexpression inhibits tumor growth, reduces metastasis, and reverses immunosuppressive TIME	Li et al. (2021a)
cEMSY	1. Binds TDP-43 to induce mitochondrial DNA leakage, activating cGAS-STING pathway and producing IFN-α/β and IL-6; 2. Releases DAMPs such as CRT and HMGB1	1. Promotes DC maturation and antigen cross-presentation, enhancing T cell activation; 2. Attracts CD8^+^T cell infiltration, synergizing with PD-1 inhibitors	1. Low expression in lung adenocarcinoma; high expression correlates with increased CD8^+^T cell and DC infiltration and improved immunotherapy response; 2. LNP-encapsulated cEMSY combined with PD-1 inhibitors induces long-term anti-tumor immunity without obvious toxicity	Zhang et al. (2025)
circ_0001946	Modulate the NER signaling pathway.	Affects the sensitivity of NSCLC cells to the chemotherapeutic drug cisplatin	Regulatory pathway suggests potential value for NSCLC immune-chemotherapy combined regimens	Zhang et al. (2021c)
circ_0000079	Inhibits ABCB1 efflux pump to reverse cisplatin resistance, indirectly promoting ICD by enhancing tumor antigen release	Indirectly activates anti-tumor immunity via ICD	Significantly downregulated in cisplatin-resistant NSCLC; overexpression restores cisplatin sensitivity, with potential value for chemotherapy- immunotherapy combined treatment	Chen et al. (2020)
circSMARCA5	1. Inhibits Wnt/β-catenin pathway to reduce chemotherapy resistance; 2. May affect T cell migration by regulating CXCL9	1. Enhances CD8^+^T cell activation and migration ability; 2. Inhibits M2 macrophage polarization	Low expression in NSCLC, associated with chemotherapy resistance; provides potential target for immunotherapy	Geng et al. (2023)
circDENND2D	Sponges miR-130b-3p to relieve inhibition on STK11, thereby inhibiting PD-L1 transcription and translation	Overexpression in A549 and H1299 cells reduces PD-L1 levels and restores CD8^+^T cell infiltration and killing activity	1. Significantly downregulated in NSCLC; low expression correlates with high PD-L1 expression and reduced CD8^+^T cell infiltration; 2. High expression correlates with prolonged PFS; synergistic tumor inhibition with anti-PD-1 therapy	Chen et al. (2023)
circFAM53B	Produces tumor-specific antigen peptides via non-canonical translation, binding HLA-I/II to activate CD8^+^/CD4^+^T cells	Low expression correlates with reduced CD8^+^T cell infiltration in NSCLC, potentially impairing anti-tumor immunity	Significantly downregulated in NSCLC; antigen-encoding property provides direction for personalized lung cancer vaccines	Huang et al. (2024b)
circCRIM1	1. Sponges miR-93/miR-182 to promote LIFR expression; 2. Competitively binds IGF2BP1 to reduce HLA-F mRNA stability	Inhibits immune escape of lung cancer cells and enhances anti-tumor activity of T cells	Low expression in NSCLC, associated with tumor progression and immune escape	Peng et al. (2023)
circMAPK1	Stabilizes CCL5 mRNA to promote CCL5 expression	Attracts CD8^+^T cell infiltration into tumor tissues and enhances anti-tumor immune response	Has potential value for improving NSCLC immune microenvironment; no clear direct clinical expression correlation	Zhao et al. (2024)

#### 4.2.1 Suppression of immune checkpoint pathways

Several circRNAs directly counteract immune checkpoint signaling. A key example is circDENND2D, the expression of which is frequently downregulated in NSCLC (Chen et al., 2023). Restoration of circDENND2D expression sponges miR-130b-3p, thereby alleviating its repression of the tumor suppressor STK11. Subsequent STK11 activation suppresses PD-L1 transcription and translation, ultimately enhancing CD8^+^ T cell infiltration and cytotoxic function (Figure 3). CircDENND2D’s ability to suppress PD-L1 and enhance CD8^+^ T cell function has been validated *in vitro* and *in vivo*, but its delivery to lung tumors remains a challenge—current *in vivo* studies use intratumoral injection, which is not feasible for advanced lung cancer.

#### 4.2.2 Promotion of sustained T cell immunity

circNDUFB2 bolsters antitumor immunity via two coordinated mechanisms: it facilitates TRIM25-mediated degradation of the oncogenic IGF2BPs proteins (Li B. et al., 2021) and serves as a ligand for RIG-I to activate the RIG-I–MAVS signaling cascade. This dual action induces the production of IFN-β and CXCL10, fostering CD8^+^ T cell recruitment and activation while simultaneously inhibiting the HLA-F-/LILRB1-mediated immune escape axis (Figure 3). CircNDUFB2’s dual mechanisms have been confirmed *in vitro* and *in vivo*, but its clinical potential is limited by the lack of data on its expression in human NSCLC subtypes. Further studies are needed to evaluate circNDUFB2’s efficacy in patient-derived xenografts and optimize its delivery to avoid activating systemic innate immune responses (e.g., RIG-I overactivation leading to inflammation).

#### 4.2.3 Activation of innate immune sensing pathways

Some circRNAs act as endogenous “danger signals” that activate pattern recognition receptors (PRRs), mimicking viral infection. For example, cEMSY (Zhang et al., 2025) (hsa_circ_0001946) binds TDP-43, inducing mitochondrial membrane depolarization and mitochondrial DNA (mtDNA) leakage into the cytoplasm. Cytosolic mtDNA then activates the cGAS–STING pathway (Zhu et al., 2021), triggering type I interferon (IFN) production and enhancing the cross-presentation of tumor antigens by dendritic cells (DCs). This, in turn, promotes the priming and activation of CD8^+^ T cells, strengthening antitumor adaptive immunity (Figure 3) Notably, cEMSY expression is downregulated in NSCLC tissues, and its restoration in preclinical models significantly inhibits tumor growth by reactivating the cGAS–STING pathway—highlighting its potential as a therapeutic target. However, the risk of excessive type I IFN production (which may cause systemic inflammation or autoimmunity) requires careful evaluation in future studies.

## 5 Potential clinical translation applications of circular RNAs in lung cancer

### 5.1 Diagnostic biomarkers

Circular RNAs (circRNAs) exhibit high stability and tissue specificity and can be detected in various body fluids of lung cancer patients (e.g., serum, plasma, and exosomes). These characteristics make circRNAs promising novel biomarkers for the early diagnosis of lung cancer. A key advantage of circRNA biomarkers over linear RNAs is their resistance to RNase degradation, which ensures reliable detection in stored samples. However, challenges remain: the lack of standardized detection methods (e.g., qPCR and RNA sequencing) and reference genes for normalization can lead to inconsistent results across studies.

For example, circRNA-002178 is highly expressed in lung adenocarcinoma (LUAD) tissues and cancer cells (Wang J. et al., 2020), and its elevated expression is also detectable in exosomes isolated from the plasma of lung cancer patients. The area under the curve (AUC) value of exosomal circRNA-002178 derived from lung cancer cells is significantly higher than that from exosomes of normal bronchial epithelial cells, supporting its potential as a non-invasive diagnostic biomarker. However, most circRNA-based diagnostic biomarkers have only been validated in small cohorts, and their performance in variable multicenter studies (e.g., differing sample protocols and detection platforms) remains unevaluated—limiting clinical translation due to unproven reproducibility.

### 5.2 Markers for drug resistance and prognosis

The expression levels of circRNAs are closely associated with the prognosis of lung cancer patients. Abnormal expression (either upregulation or downregulation) of specific circRNAs correlates with poor clinical outcomes, enabling their use as independent prognostic indicators.

For instance, circCRIM1 expression is downregulated in non-small cell lung cancer (NSCLC) (Peng et al., 2023), and its low expression is associated with unfavorable prognosis, suggesting its role as a tumor suppressor and a prognostic biomarker. In contrast, circIGF2BP3 is highly expressed in NSCLC and correlates with poor survival, serving as an independent prognostic factor (Liu et al., 2021). While these prognostic correlations are observed in retrospective studies, prospective validation (e.g., following patients for 5 years to assess overall survival) is lacking for most circRNAs.

CircRNAs can also indicate drug resistance. In cisplatin (DDP)-resistant NSCLC cells, the expression of hsa_circ_0000190 is significantly upregulated (He and Li, 2024). It sponges miR-1253 (Wang Y. Q. et al., 2020), alleviating its repression of IL-6 transcription, which promotes IL-6 expression and contributes to chemoresistance. Additionally, exosomal circUSP7 derived from tumor cells induces CD8^+^ T cell dysfunction and confers resistance to anti-PD-1 therapy in NSCLC via the miR-934/SHP2 axis (Chen S. W. et al., 2021). However, the mechanism by which circRNAs predict drug resistance (e.g., circ_0000190 in cisplatin resistance) is often validated *in vitro* but not in patient-derived organoids, which better recapitulate clinical drug responses.

### 5.3 Immunotherapeutic targets

Given the critical roles of circRNAs in regulating the lung cancer immune microenvironment, they are emerging as promising therapeutic targets. Targeting circRNAs may remodel the immune microenvironment, enhance anti-tumor immunity, and improve treatment outcomes. Moreover, circRNA-based vaccines and circRNA-enhanced chimeric antigen receptor T (CAR-T) cell therapy represent novel strategies—either through encoding tumor-associated antigens or immunomodulatory molecules to potentiate anti-tumor immune responses (Table 6).

**TABLE 6:** Basic and clinical research on inhibiting lung cancer using immunomodulatory effects of CircRNAs.

Research phase	circRNA/Therapy name	Mechanism of action	Research data	Clinical progress	References
Clinical Phase	Intranasal circRNA Vaccine (LNP-encapsulated)	1. Uses LNPs to encapsulate circRNAs encoding antigen epitope peptides (e.g., SIINFEKL), 2. Relies on tumor antigens and type I conventional dendritic cells for action; AMs enhance antigen-specific T cell response in lung tissues; 3. Antigen-specific T cells are initially activated in extrapulmonary draining lymph nodes, directly enhancing T cell response in lung tissues and activating pulmonary immune system	1. Prevention mode: 70% of lung cancer model mice achieved long-term tumor-free survival; 2. Treatment mode: Tumor burden reduced by 83%, median survival extended from 18 days to 35 days	International patent applied; human clinical trial initiated in 2024, enrolling patients with advanced NSCLC	Li et al. (2025)
Clinical Phase (Translational)	LNP-circRNA Drug Encoding IL-12	1. Delivers circRNAs encoding IL-12 to lung tumors via H1L1A1B3 LNP vector; 2. IL-12 activates APCs, stimulates production of cytokines such as IFN-γ, and enhances anti-tumor immune response of T cells and NKT cells	1. In LLC mouse models, single tumor injection or intratracheal administration significantly inhibited tumor progression; 2. Combined use with ICIs significantly induced tumor regression	In translational progress; human clinical trial not yet clearly initiated	Xu et al. (2024)
Preclinical Research	cEMSY (ICD Inducer)	1. Interacts with RNA-binding protein TDP-43 to induce its mitochondrial accumulation, causing mitochondrial DNA leakage, activating cGAS-STING pathway, and producing anti-viral immune response; 2. Induces immunogenic cell death in lung adenocarcinoma cells, releases DAMPs, and promotes cross-activation of T cells by dendritic cells	1. Intratumoral delivery of LNP-encapsulated cEMSY induces strong anti-tumor immune response in immunosuppressive lung cancer models; 2. Intratumoral administration of cEMSY-LNP significantly sensitizes lung adenocarcinoma mouse models to anti-PD-1 therapy	Preclinical research phase; completed *in vitro* and *in vivo* verification of ICD-inducing ability and combined effect with PD-1 blockade therapy	Zhang et al. (2025)
Preclinical Research	circRNA-Mediated anti-DLL3 CAR-T Therapy	Delivers circRNAs encoding anti-DLL3 CAR to human primary T cells to construct anti-DLL3 CAR-T cells, which target and kill small cell lung cancer cells expressing DLL3	1. In SCLC mouse models, tail vein injection of this CAR-T cell showed stronger tumor-killing effect; 2. Higher safety and more convenient *in vivo* delivery potential compared with traditional viral vector CAR-T methods	verified *in vitro* and *in vivo* tumor-killing effect and safety	Cai et al. (2025)
Preclinical Research	circDENND2D	1. Significantly downregulated in NSCLC; sponges miR-130b-3p to relieve inhibition on serine/threonine kinase STK11; 2. Further inhibits transcription and translation of PD-L1, reduces PD-L1 expression on tumor cell surface, and restores CD8^+^T cell infiltration and killing activity	1. Overexpression of circDENND2D significantly reduced PD-L1 protein level; 2. In clinical samples, low expression of circDENND2D correlated with high PD-L1 expression and reduced CD8^+^T cell infiltration	Preclinical research phase; verified cell-level mechanism and clinical sample correlation	Chen et al. (2023)
Preclinical Research	circPLEKHM1	Promotes M2 macrophage polarization in NSCLC, thereby driving tumor metastasis; targeting circPLEKHM1 can reverse this polarization and inhibit formation of immunosuppressive microenvironment	In mouse models, therapeutic strategies targeting circPLEKHM1 showed potential to inhibit lung cancer metastasis and improve tumor immune microenvironment	Preclinical research phase; verified anti-metastatic effect and immune microenvironment regulation effect	Wang et al. (2024)
Basic Research	circ-HSP90A	1. Recruits USP30 to stabilize HSP90A, stimulating the STAT3 signaling pathway; 2. Sponges miR-424-5p to regulate PD-L1 expression and participate in tumor immune escape	Knockdown of circ-HSP90A inhibited proliferation, migration, and invasion of NSCLC cells, and reduced immune escape-related phenotypes	Basic research phase	Lei et al. (2023)
Basic Research	circ_0000052	Sponges miR-382-3p to relieve inhibition on PD-L1, upregulates PD-L1 expression, and promotes immune escape of NSCLC cells	-	Basic research phase	Zhang et al. (2023)
Basic Research	m6A-Modified circZNF548	Regulates exosomal miR-7108-3p, activates CD3^+^CD8^+^T cell activity dependent on JMY protein, enhances the anti-tumor function of CD3^+^CD8^+^T cells, and thereby inhibits NSCLC growth	-	Basic research phase	Zhao et al. (2025)

#### 5.3.1 circRNA vaccines: precise immune activation

The key advantage of circRNA vaccines lies in their molecular stability. Unlike linear mRNAs, circRNAs lack 5′ caps and 3′ poly(A) tails, conferring strong resistance to exoribonuclease degradation and enabling sustained antigen expression. A notable example is an intranasal lipid nanoparticle (LNP)-encapsulated circRNA vaccine developed by a team from Tsinghua University (Li H. et al., 2022). Delivered via the nasal mucosa, this vaccine leverages the unique immune environment of the lung (Xu et al., 2023): antigen-presenting cells (APCs), especially cDC1 subsets, efficiently take up antigens and migrate to draining lymph nodes, thereby activating antigen-specific CD8^+^ T cells. Simultaneously, alveolar macrophages amplify T cell responses by secreting cytokines such as IL-12 and IFN-γ, enhancing local immunity.

Notably, this circRNA vaccine induces tissue-specific immunity. Intranasal immunization preferentially generates lung-resident memory T cells (Trm), which are critical for preventing lung cancer recurrence. Preclinical studies showed that a single dose resulted in antigen expression lasting over 30 days, with neutralizing antibody titers significantly higher than those induced by linear mRNA vaccines (p < 0.01) (Thornhill-Wadolowski et al., 2025).

This vaccine has entered Phase I clinical trials to evaluate safety and immunogenicity in patients with advanced NSCLC (Huang G. et al., 2024)—key remaining steps include assessing long-term efficacy (e.g., 1-year progression-free survival), optimizing LNP targeting to lung APCs, and reducing potential local side effects (e.g., nasal inflammation).

#### 5.3.2 circRNA-enhanced CAR-T therapy: overcoming solid tumor challenges

CAR-T therapy in solid tumors is limited by T cell exhaustion and on-target/off-tumor toxicity. CircRNAs can prolong CAR expression and improve T cell persistence. A research team from Peking University used *in vitro*-transcribed circRNA to encode an anti-DLL3 CAR and introduced it into human primary T cells via electroporation (Jaspers et al., 2023). Compared to linear mRNA-transfected T cells, circRNA-modified T cells showed extended CAR surface expression and a 40% reduction in peak secretion levels of cytokines associated with cytokine release syndrome (CRS) (e.g., IL-6 and IFN-γ).

Mechanistically, the circular structure minimizes aberrant activation of innate immune sensors (e.g., RIG-I), reducing T cell exhaustion (Iurescia et al., 2020; Ren et al., 2024). In a small cell lung cancer (SCLC) mouse model, circRNA-CAR-T treatment increased tumor-infiltrating T cells (Lasvergnas et al., 2022), decreased exhausted T cells (PD-1^+^TIM-3^+^) by 50%, and significantly extended median survival. This approach provides a new strategy for developing controllable and durable CAR-T therapies.

#### 5.3.3 circRNA-mediated regulation of immune checkpoints: from mechanism to therapy

Multiple studies show that circRNAs can regulate immune checkpoint molecules through competing endogenous RNA (ceRNA) mechanisms. For example, circDENND2D is downregulated in LUAD and functions as a sponge for miR-130b-3p (Chen et al., 2023), relieving its inhibition of the tumor suppressor STK11 (LKB1). STK11 activates the AMPK pathway, which suppresses mTOR-mediated PD-L1 transcription. Restoration of circDENND2D reduced the proportion of PD-L1^+^ tumor cells from 65% to 22% and increased CD8^+^ T cell infiltration.

Another circRNA, cEMSY (hsa_circ_0001946), recruits TDP-43 to mitochondria (Zhang et al., 2025), inducing membrane depolarization and mtDNA leakage into the cytoplasm. Cytosolic mtDNA activates the cGAS–STING pathway, stimulating type I interferon production and immunogenic cell death (ICD). In a Kras^+^/p53^-^ mouse model, intratumoral injection of cEMSY-loaded LNPs increased the response rate to anti-PD-1 therapy from 20% to 80% (Hu et al., 2025). This approach remains in preclinical stages. Key steps before clinical application include scaling up circRNA production for GMP compliance, testing in patient-derived xenografts of SCLC, and evaluating on-target/off-tumor toxicity in non-human primates.

#### 5.3.4 Novel therapeutic paradigm: circRNAs encoding immunomodulatory factors

A team from the University of Toronto developed an LNP-formulated circRNA encoding IL-12 (circIL12) (Xu et al., 2024). By optimizing the internal ribosomal entry site (IRES) and open reading frame (ORF), they achieved 3.8-fold higher protein expression in lung epithelial cells compared to linear mRNA.

In a Lewis lung carcinoma (LLC) model, intratracheal delivery of circIL12 induced local IFN-γ concentrations up to 350 pg/mg and recruited NK cells and effector memory T cells (Kim et al., 2021). When combined with anti-PD-L1 therapy, the complete response (CR) rate reached 60%, without observed systemic IL-12-related toxicity. This strategy is in preclinical stages, with key steps including validating protein expression in human lung epithelial cells, optimizing intratracheal delivery devices for clinical use, and testing in patient-derived xenografts of NSCLC. Additionally, long-term safety needs to be evaluated in large animal models.

## 6 Challenges in circRNA research on lung cancer immune microenvironment

### 6.1 Complexity of mechanistic elucidation and functional validation

Research on the functions of circRNAs in the lung cancer TIME is still in its early stages. Their circular structure and low abundance pose significant technical challenges for detection. For example, conventional RNA sequencing struggles to accurately capture circRNAs and often requires RNase R pretreatment or specific primer design. Even with single-cell sequencing or spatial transcriptomics, issues such as insufficient sensitivity and algorithm optimization remain. Furthermore, circRNAs often function as “molecular sponges” that sequester miRNAs or interact with RBPs to regulate immune-related signaling pathways (e.g., NF-κB and cGAS–STING) (Peng et al., 2025; Zhong et al., 2024). However, functional validation is challenging: knocking down a circRNA may affect its linear homologous gene, and tools like CRISPR-Cas13 still carry off-target risks (Neisewander, 2022). The involvement of cross-cell-type regulatory networks—such as circRNA transmission between tumor cells and macrophages via exosomes—further increases the complexity and difficulty of mechanistic studies (Zhu et al., 2025). A critical gap is the lack of *in vivo* validation for many circRNA mechanisms. For instance, circSHKBP1’s role in M2 polarization is primarily confirmed *in vitro*, and its impact on the TIME in animal models is unknown. This limits the reliability of their therapeutic potential as *in vitro* conditions do not fully replicate the human lung cancer microenvironment.

### 6.2 Dual limitations: delivery efficiency and immunogenicity

The clinical translation of circRNA-based therapies urgently requires solutions to the insufficient targeting efficiency of delivery systems. Existing LNPs exhibit low enrichment rates in lung tumors (approximately 15%) and poor penetration into deep tumor tissues. Although intranasal delivery of circRNA vaccines can activate mucosal immunity, their penetration into solid tumors remains limited. On the other hand, circRNAs may activate nonspecific immune responses through pattern recognition receptors (e.g., TLR3/7) (Xu et al., 2025; Luan et al., 2024), leading to inflammatory reactions or organ toxicity. Balancing immune activation and safety is a major hurdle that must be overcome for circRNA vaccines and therapies to enter clinical application. The intranasal circRNA vaccine in Phase I trials faces challenges in penetrating large lung tumors, and systemic delivery of LNPs may cause liver accumulation. Key steps to address this include engineering lung-targeted LNPs (e.g., modifying surface ligands to bind lung epithelial cell receptors) and testing their biodistribution in non-human primates.

### 6.3 Unresolved issues: dynamic regulation and drug resistance mechanisms

The expression of circRNAs in the TIME is regulated by various microenvironmental factors (e.g., hypoxia and inflammation), yet the dynamics of their expression and their causal relationship with immune cell function remain unclear. For instance, while circZNF451 promotes M2 macrophage polarization via exosomes, the regulatory mechanisms of its upstream factors (e.g., HIF-1α) require further investigation (Thomas et al., 2022). Additionally, circRNAs play dual roles in immunotherapy resistance: some (e.g., circ_0000079) can enhance chemosensitivity (Ma et al., 2024), whereas others (e.g., circ_002178) induce immune resistance through the PD-L1/PD-1 pathway. These context-dependent mechanisms urgently need systematic elucidation.

### 6.4 Key contradictions in circRNA-mediated immunoregulation

A central debate concerns their context-dependent functionality, where identical circRNAs demonstrate opposing roles across studies. This duality, exemplified by hsa_circ_0000190, likely stems from divergent functions within different cellular compartments of the tumor microenvironment (He and Li, 2024; Yang et al., 2022; Chen et al., 2017).

The canonical “miRNA sponge” mechanism is increasingly challenged by stoichiometric inefficiency, shifting focus toward protein-centric functions. CircRNAs are now implicated in direct protein interactions (e.g., circ-CPA4 modulating PD-L1) and potential translation, though robust *in vivo* validation remains scarce.

Many circRNAs detected in the tumor microenvironment or patient blood remain elusive, and their cell-type specificity is poorly characterized. Furthermore, the regulatory mechanisms governing selective packaging of circRNAs into exosomes for intercellular communication are not well defined.

## 7 Future prospects and technological breakthroughs

### 7.1 Technological innovations for mechanistic insights and precision medicine

Next-generation detection technologies, such as single-cell circRNA sequencing and spatial transcriptomics, are expected to reveal the distribution and functional heterogeneity of circRNAs within cellular subpopulations in the TIME. Combined with high-throughput techniques like CLIP-seq and RIP-seq, these approaches can help construct circRNA–immune interaction networks, providing a basis for target screening. Moreover, exosomal circRNAs (e.g., circPLEKHM1) have shown great clinical potential as liquid biopsy biomarkers in lung adenocarcinoma (Wang et al., 2024).

### 7.2 Engineering innovations in delivery systems

Optimizing LNPs for lung tissue targeting (e.g., customized H1L1A1B3 formulations), engineering exosomal vectors (e.g., surface modification with targeting ligands), and developing novel cyclization systems (e.g., Group I intron-based self-splicing circularization) will significantly enhance the delivery efficiency and stability of circRNAs. Combination delivery strategies—such as co-encapsulating circRNAs with ICIs or chemotherapy drugs—may synergistically enhance anti-tumor immunity and overcome drug resistance.

### 7.3 Diversified pathways for clinical translation

Combining circRNA vaccines (e.g., intranasally delivered LNP-circRNA) (Wan et al., 2024) with immune checkpoint inhibitors has achieved complete tumor regression with manageable toxicity in preclinical studies. circRNA-based CAR-T therapies (e.g., DLL3-targeting circRNA-CAR-T) (Cai et al., 2025) demonstrate more sustained anti-tumor activity and improved safety profiles. Additionally, antisense oligonucleotides (ASOs) or CRISPR-Cas13 systems can precisely target oncogenic circRNAs (e.g., circPHLPP2) (Hu et al., 2024) to reverse the immunosuppressive microenvironment.

## 8 Conclusion

CircRNAs regulate the lung cancer immune microenvironment through diverse molecular mechanisms, including modulating immune checkpoint molecules, influencing immune cell infiltration and function, and regulating inflammatory responses and immunosuppressive microenvironments. These mechanisms provide new targets and strategies for lung cancer immunotherapy. A deeper understanding of the molecular mechanisms by which circRNAs regulate the lung cancer TIME is crucial for developing novel circRNA-based diagnostic markers, prognostic indicators, and therapeutic targets. Critically, therapeutically targeting circRNAs—using tools such as CRISPR-Cas13 or antisense oligonucleotides—is becoming increasingly feasible and holds significant promise for overcoming immunotherapy resistance. This is supported by emerging preclinical and early clinical efforts, such as intranasal circRNA vaccines and circRNA-enhanced CAR-T therapies, which aim to remodel the immunosuppressive TIME. Although many challenges remain in circRNA research, continued technological advances and in-depth investigations hold great promise for the application of circRNAs in precision therapy for lung cancer.
